# *De novo* p.Glu61Ter mutation in GCH1 in a Moroccan patient with dopa-responsive dystonia: a case report

**DOI:** 10.11604/pamj.2024.47.159.36397

**Published:** 2024-04-03

**Authors:** Ahmed Bouhouche, Leila Tamaoui, Nazha Birouk

**Affiliations:** 1 Laboratory of Human Genetics, Medical School and Pharmacy, University Mohammed V, Rabat, Morocco,; 2 Department of Neurology and Neurogenetics, Specialties Hospital, CHU Ibn Sina, Rabat, Morocco,; 3 Department of Neurophysiology, Specialties Hospital, Rabat, Morocco

**Keywords:** Dopa-responsive dystonia, GCH1 gene, *de novo* non-sense mutation, case report

## Abstract

Dopa-responsive dystonia (DRD) is a hereditary movement disorder due to a selective nigrostriatal dopamine deficiency. It is characterized by onset in childhood or adolescence with marked diurnal fluctuation with or without Parkinsonian features, and is caused by mutations in GCH1 gene. We report in this study the clinical and genetic features of the first DRD Moroccan patient. Using a gene panel sequencing, we identified a heterozygous nonsense variant p. Glu61Ter in GCH1. A subsequent targeted segregation analysis by Sanger sequencing validated the presence of the mutation in the patient, which was found to have occurred de novo. The objective of this study is to report the first description of DRD in Morocco, and highlights the importance of new generation sequencing technology in the reduction of medical wandering and the management of hereditary diseases.

## Introduction

Dopa-responsive dystonia (DRD) was first reported by Segawa *et al*. [1]. It is caused by heterozygous mutations in the *GCH1* gene located on chromosome 14q22.2 and coding for the GTP cyclohydrolase I enzyme involved in the biosynthesis of tetrahydrobiopterin (BHA) which acts as a cofactor in the production of dopamine and serotonin [2]. The clinical characteristics of DRD are childhood- or juvenile-onset dystonia with marked diurnal fluctuation with or without Parkinsonian features. The excellent and sustained efficacy of low dose of L-Dopa without motor fluctuations or dyskinesia represent the hallmark of the disease [3]. More than 130 mutations have been associated with DRD, including single-nucleotide variations with missense, nonsense, splice and indels mutations, as well as large structural mutations with deletions of exons or the entire gene [3], whereas *de novo* mutations are rare and poorly documented [4]. Dopa-responsive dystonia patients have been reported in all ethnicities, but rarely in the African continent, where only a few *GCH1* mutations have been identified [5,6] due to the lack of studies and not that of patients. Here, we report the first description of DRD in a patient from Morocco, with a *de novo* nonsense mutation p.Glu61Ter in *GCH1* identified by gene panel sequencing.

## Patient and observation

**Patient information:** a 16-year-old man has had a history of gait abnormality since he was 9. He complained of falls while running or after walking one meter associated with head tilted forward and slowness of movements. He described that his symptoms were slight in the morning and progressively worsened by the end of the day and after an effort.

**Clinical findings:** neurological examination revealed a gait difficulty with knees flexed, adduction of thighs and body bent forward. Bradykinesia, rigidity and brisk patellar and Achilles reflexes were also observed. No tremor or abnormal signs in the upper limbs and face was seen. The remaining findings were consistent with intact cranial nerve and normal muscle strength, cerebellar and sensory functions.

**Diagnostic assessment:** brain magnetic resonance imaging was unremarkable.

**Therapeutic intervention:** the patient received a dose of 62.5 mg of Levodopa twice daily.

**Follow-up:** the patient's symptoms improved dramatically. He did no longer fall and recovered a nearly normal gait and running capacity following several days after initiation of Levodopa. A sustained effect was obtained without side effects until now after 13 years of follow-up.

**Diagnosis:** a clinical diagnosis of DRD was made considering his early-onset dystonia which fluctuates throughout the day and significantly improves with levodopa treatment.

**Family history:** the patient´s father and sister (II.6 and III.2) developed slight action hands tremor by the age of 50 and 13 respectively. Their symptoms didn´t respond to Levodopa but have partially improved using propranolol 60 mg/d. The patient's grandmother and uncle seemed to present essential tremor features according to patient's statement. Interestingly, no family member exhibit features suggestive of dystonia.

**Figure 1:**
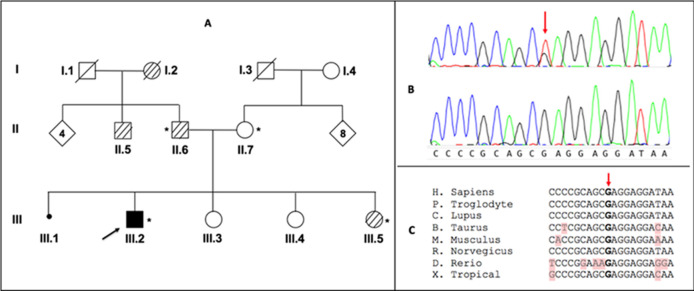
pedigree of the studied family (A); darkened square indicates the patient with dopa-responsive dystonia and hatched squares and circles indicate males and females with essential tremor; arrow: index patient; asterisk: genetic testing performed; sanger sequencing confirms the presence of the c.181G>T mutation in GCH1 in heterozygous state (B); partial nucleotide sequence alignment of human GCH1 with orthologs shows evolutionary conservation between species of the nucleotide 181 and the codon 61 (C)

**Genetic analysis:** blood samples were taken from the patient III.2 and family members II.6, II.7 and III.5 (Figure 1A), and genomic DNA was extracted using Wizard® Genomic DNA Purification Kit from Promega. The DNA samples were then analyzed by next-generation sequencing using a gene panel designed for parkinsonism and overlapping phenotypes according to the protocol already described [7]. Variant analysis and annotations were done using Ion Reporter Software v15 (Thermo Fisher Scientific). A heterozygous c.181G>T mutation (NM_000161.2) was identified in exon 1 of the *GCH1* gene leading to a replacement of glutamine at position 61 in the protein by a stop codon (p.Glu61Ter). A subsequent targeted segregation analysis by Sanger sequencing validated the presence of the mutation in the patient (Figure 1B) and its absence in the parents, confirming that the mutation has occurred *de novo*. The *GCH1* c.181G>T variant was absent from the 1000 genome project and the gnomAD databases, and it was completely absent in 192 ethnically matched chromosomes. This *de novo* nonsense variant affects a highly conserved amino acid (Figure 1C) and is classified as pathogenic according to the ACMG guidelines.

**Patient perspective:** the patient was satisfied with his treatment which did not cause him any side effects. Genetic counseling was offered to the family for the risk of transmission of this hereditary disease.

**Informed consent:** all family members and their legal representatives gave written informed consent to the study.

## Discussion

Dopa-responsive dystonia is an autosomal dominant neurodegenerative disorder with a highly heterogeneous clinical phenotype. The classic clinical features are a childhood or adolescent-onset foot dystonia characterized by diurnal fluctuations with symptoms aggravating by the afternoon and disappearing after sleep. This focal dystonia may gradually progress to other parts of the body and may be associated with some features of parkinsonism [1]. However, the clinical picture has been reported to be heterogeneous and can vary from asymptomatic cases to full features of dystonia [2].

The clinical phenotype of the studied patient strongly evoked DRD due to mutations in *GCH1*. Next-generation sequencing using Ion proton technology with a panel of 20 genes designed for Parkinsonism and related disorders found a heterozygous nonsense mutation c.181G>T (p.Glu61Ter) in exon 1 of *GCH1* in the patient. The mutation was validated by Sanger sequencing in the patient, and was not found in the parents confirming the diagnosis of DRD in the patient studied and the *de novo* nature of this variant. Interestingly, the family history showed in four family members the presence of essential tremor, unrelated to DRD since the mutation was absent in the tested patient's sister and father. The exome sequencing carried out in the father was negative for all known genes responsible for essential tremor and for the 8000 hereditary diseases known so far.

The identified *GCH1* c.181G>T variant was considered first novel after the data annotation since it has not a dbSNP identifier and was absent from the 1000 genome project, the gnomAD and the Clinvar databases. However, this mutation has long been reported in a genetic database of dystonia and parkinsonism (mdsgene.org). Furthermore, it was recently reported as recurrent by Weissbach A *et al*. [3] since it has been documented in eight families.

The most classic molecular pathophysiology of DRD refers to biopterin deficiency, leading further to dopamine decrease [2]. Accordingly, and as in the present case, patients exhibit a complete and sustained resolution of symptoms related to DRD with a low dose of Levodopa therapy without motor complications after many years with this agent. Nevertheless, several cases with proven *GCH1* mutations show residual motor symptoms [8]. Therefore, it was recommended that a levodopa trial be instituted as the primary differential diagnosis in every patient with suspected DRD [9].

## Conclusion

We report in this study the first clinical and molecular description of a Moroccan patient with DRD. The p.Glu61Ter mutation identified in *GCH1* is recurrent since it has been reported in several families, but it occurred *de novo* for the first time in our patient.
